# Metabolic Alterations in Crassostrea Gigas After Feeding Selenium-Enriched Yeast Based on Transcriptomic Analysis

**DOI:** 10.3390/biology14070898

**Published:** 2025-07-21

**Authors:** Yancheng Zhao, Xiaojing Jiang, Liming Jiang, Yongjie Wang, Cuiju Cui, Xiumei Liu, Zan Li, Weijun Wang, Jianmin Yang

**Affiliations:** 1 School of Fisheries, Ludong University, Yantai 264025, China; zycheng6633@163.com (Y.Z.); wyj2214487079@126.com (Y.W.); cuicuiju@163.com (C.C.); ladderup@126.com (J.Y.); 2 College of Life Sciences, Yantai University, Yantai 264005, China; xiumei0210@163.com; 3 Yantai Campus, Binzhou Medical University, Yantai 264003, China; jiangxiaojing@bzmc.edu.cn; 4 Yantai Marine Economic Research Institute, Yantai 264003, China; ytlmjiang@163.com

**Keywords:** *Crassostrea gigas*, transcriptomic, selenium-enriched yeast, protein-protein interaction network

## Abstract

This study explores the effects of selenium-enriched yeast supplementation on growth-related and immune-related gene expression in *C. gigas*, aiming to support feed optimization in oyster aquaculture. Selenium, an essential trace element, is vital for growth, immune function, and metabolism in animals. Selenium-enriched yeast, an organic form, offers superior bioavailability, enabling efficient absorption and utilization. *C. gigas*, a commercially significant marine shellfish, is rich in protein and nutrients, but the effects of selenium on mollusks remain insufficiently explored. In this study, oysters were divided into three groups: a control group without selenium (THNP), a 2 ppm selenium group (THMP), and a 4 ppm selenium group (THHP). Transcriptome sequencing yielded 388,679,026 clean reads. GO and KEGG enrichment analyses identified key metabolic signaling pathways, and a PPI analysis was performed on the translation products of DEGs involved in the KEGG pathways. qRT-PCR validated the expression of principal DEGs. The combined results of enrichment and PPI analyses highlighted pathways such as glutathione metabolism and collagen signaling. Additionally, three hub genes—*FASN*, *HRAS*, and *ABCG5*—were identified as central to the selenium response. These findings enhance the understanding of selenium’s molecular impact on oysters and support its application in aquaculture.

## Simple Summary

This study revealed that supplementing *C. gigas* feed with selenium-enriched yeast significantly alters gene expression related to metabolism, immunity, and tissue structure. Specifically, moderate selenium addition (2 ppm) enhanced metabolic activity and promoted fattening, while higher concentrations (4 ppm) may induce immune responses. These findings suggest that appropriate selenium supplementation can improve oyster growth and quality, offering a promising strategy to boost efficiency and economic returns in oyster and broader shellfish aquaculture industries.

## 1. Introduction

Selenium is an essential dietary trace element that plays a critical role in the growth, immune response, and metabolic processes of humans and animals [1]. In nature, selenium exists in two primary forms: the hexavalent cation of selenate (Se^6+^) and the tetravalent cation of selenite (Se^4+^) [2]. As it can exist in the form of selenocysteine within selenoproteins, selenium is involved in various biological pathways, enhancing antioxidant capacity and exerting anti-inflammatory and antimicrobial effects [3,4]. Plants, capable of absorbing selenium from the soil, do not require this trace element for survival [5]. Selenium deficiency in animals leads to a plethora of adverse effects, such as weakened immune systems, metabolic abnormalities, and hindered growth and development [6,7,8]. Previous studies have shown that feeding tilapia with selenium-rich diets significantly promotes growth, increases the activity of antioxidant enzymes, and enhances the transcription levels of immune-related proteins [9]. Research by Tian et al. has revealed that elemental selenium (Se) significantly increases the expression of the IFN and ISG15 genes in EPC cells of crucian carp and zebrafish, thus strengthening their immune defense [10]. Compared to inorganic selenium, selenium-enriched yeast, known for its high absorbability and effectiveness, is more widely used as a feed additive [11]. While studies on selenium’s impact on immunity and metabolism are more common in vertebrates, such research is yet to be conducted in mollusks. Meanwhile, selenium-enriched yeast, an important form of selenium supplementation, is characterized by high absorption and low biotoxicity compared to other inorganic selenium [12]. Hence, this study utilizes selenium-enriched yeast as a dietary selenium supplement to investigate its effects on biological processes such as metabolism and immunity in organisms. It has been suggested that inorganic selenium accumulation in organisms affects their reproductive function, but further research is needed on the bioaccumulation of organic selenium [13,14].

*C. gigas* is one of the widely cultivated marine mollusks across the globe [15]. As a high-quality protein source among seafood, the demand for oysters has significantly risen [16]. Therefore, the question of whether selenium supplementation in feed can enhance the growth and metabolism of *C. gigas* remains to be addressed and is a direction for research aimed at increasing oyster aquaculture yields.

High-throughput transcriptome sequencing technology, also known as “next-generation” sequencing technology, is a highly efficient and rapid method for sequencing [17,18,19]. This technology enables the parallel sequencing of a large number of nucleic acid molecules simultaneously, generating extensive data. In recent years, transcriptome sequencing has been extensively utilized in the study of biological mechanisms. For instance, Ding and colleagues employed transcriptome sequencing to analyze the key genes determining the different shell colors of scallops, enhancing our understanding of the molecular mechanisms involved in the growth, immunity, shell pigmentation, and biomineralization of the species [20]. In our study, transcriptome sequencing was employed to investigate the molecular mechanisms underlying the response of *C. gigas* following dietary supplementation with selenium-enriched yeast. The findings of this study demonstrate the promotional effects of selenium on the growth and development of *C. gigas*, providing a reference for selenium supplementation in oyster aquaculture feeds.

## 2. Materials and Methods

### 2.1. Sample Collection

All the oyster samples required for this experiment (Total weight = 50 ± 20 g) were sourced from the seas near Yantai, Shandong, China, and acclimatized in breeding pools for one week to fully adapt to the experimental seawater conditions (dissolved oxygen = 5.4 mg/L, pH = 8.1, salinity = 30 ± 0.5 ppt). Three 6000 L pools with identical seawater parameters were set up, each holding 30 oysters. All the pools were fed six times daily. In the control group, 24 g of yeast was fed once daily, and live diatoms were fed during the remaining five feedings. In the 2 ppm treatment group, 12 g of yeast and 12 g of selenium-enriched yeast were fed once daily, with live diatoms provided during the other five feedings. The selenium content of the selenium-enriched yeast used was 2000 mg/kg (dry weight; Angel Nutritech, China). In the 4 ppm treatment group, 24 g of selenium-enriched yeast was fed once daily, along with five feedings of live diatoms. For each treatment group, the respective yeast or selenium-enriched yeast was dissolved into 12 L of feed to ensure consistent selenium delivery and uniform distribution [21]. The pools were changed daily to ensure that the concentration was constant. After 45 days of the experiment, which began in June 2023, nine oysters were randomly selected from each experimental group, and tissues from the hepatopancreas were collected. Total RNA was individually extracted from each oyster, and equal molar amounts of RNA from every three individuals were pooled to construct one sequencing library, resulting in three RNA-seq libraries (technical replicates) per group (Table S1). Once sample extraction was completed, they were immediately stored in cryotubes and frozen in liquid nitrogen for subsequent transcriptome sequencing.

### 2.2. RNA Extraction, Library Construction, and Sequencing

The RNA extraction, library construction, and sequencing for this study were technically supported by Beijing Novogene Corporation (Beijing, China). The Trizol method was used to extract RNA from the hepatopancreas samples, with quality control supported by the Agilent 2 100 Bioanalyzer (Agilent Technologies, Santa Clara, CA, USA). The NEBNext^®^ Ultra™ Directional RNA Library Prep Kit (New England Biolabs, Ipswich, MA, USA) was employed for library construction. The sequencing was conducted on the Illumina NovaSeq 6 000 platform (Illumina, Inc., San Diego, CA, USA) [22].

### 2.3. Differential Gene Identification

The raw data obtained from sequencing were cleaned. Reads containing adapters, those with a N ratio greater than 10%, and low-quality reads (with a Qphred quality value ≤ 20 and base count over 50) were all removed, constituting a percentage of the total reads. The clean reads were mapped to the *C. gigas* reference genome vN1 (GCA_011032805.1), which has been deposited in NCBI. The corresponding annotation file used for gene mapping and functional analysis is not yet publicly available. DESeq2 (v1.38.3) in R (v4.2.2) was employed to identify Differentially expressed genes (DEGs) meeting the criteria of |Foldchange| > 1.5 and a *p*-value ≤ 0.05 [23]. Volcano plots, Venn diagrams, and cluster heatmaps were used to display the differential gene expression in oysters fed with selenium-supplemented diets.

### 2.4. Enrichment Analysis of DEGs

DAVID (v6.8) was utilized for the functional enrichment analysis of the identified DEGs (Sherman et al., 2022 [24]). The analysis was conducted using the default parameters (Count = 2, EASE = 0.1) of the website. The background gene set used for enrichment analysis consisted of all the expressed genes identified in the transcriptome data prior to differential expression filtering [25].

### 2.5. Analysis of Protein–Protein Interaction Networks

The STRING v12.0 online platform was employed to construct the protein–protein interaction (PPI) network [26]. The gene sequences of all the DEGs were uploaded to STRING for homologous sequence alignment, and the sequences with the highest scores were used for STRING analysis. The minimum interaction score was set at 0.150, with the other parameters retained as default. The role of key DEGs following organic selenium supplementation in feed was analyzed based on the number of involvements in Kyoto Encyclopedia of Genes and Genomes (KEGG) pathways and PPI.

### 2.6. Quantitative RT-PCR Verification

Finally, twelve DEGs with key roles in oysters after the addition of organic selenium were used to characterize the accuracy of sequencing. The accuracy of the sequencing results was demonstrated by the concordance between the expression trends of the genes in the sequencing results and the quantitative Reverse Transcription Polymerase Chain Reaction (qRT-PCR) results [27,28]. The Primer Premier 5.0 software was used to design specific primers for quantitative validation (Table 1). Due to the stability of expression, EF-1α was used as a housekeeping gene. The 2^−ΔΔCT^ method was used to calculate the relative amount of fold change in expression of the target gene over the housekeeping gene.

## 3. Results

### 3.1. Sequencing Quality

High-throughput sequencing technology was applied to sequence oyster hepatopancreas samples. A total of 388,679,026 usable reads were generated for subsequent analysis. Over 67% of the clean reads could be mapped to the reference genome, with a quality score of 95% Q20 and 92% Q30 (Table 2). These high-quality data attest to the reliability of the subsequent analyses [29].

### 3.2. Differential Gene Expression Analysis

**Figure 1:**
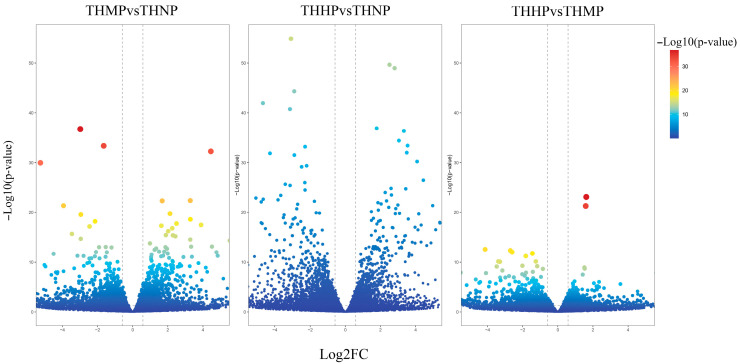
Volcano plot of DEG expression after the addition of organic selenium to the feed. The *x*-axis represents the log2 Fold change; the *y*-axis indicates the change in *p*-value. Each point in the graph represents a gene, with the color transition from blue to red indicating the variation in the *p*-value of the gene.

In this study, we utilized volcano plots to display the differential expression of DEGs in oysters fed with diets supplemented with organic selenium (Figure 1).

Compared to the control group (THNP), the treatment group with 2 ppm selenium (THMP) exhibited 1144 upregulated DEGs and 1533 downregulated genes. In the comparison between the THNP group and the treatment group with 4 ppm selenium (THHP), 1533 DEGs were upregulated, and 2179 DEGs were downregulated. Between the THMP group and the THHP group, 514 genes were significantly upregulated, and 847 DEGs were significantly downregulated. A large number of significantly DEGs were identified in oysters fed organic selenium-enriched yeast, indicating substantial transcriptomic changes in response to selenium treatment.

**Figure 2:**
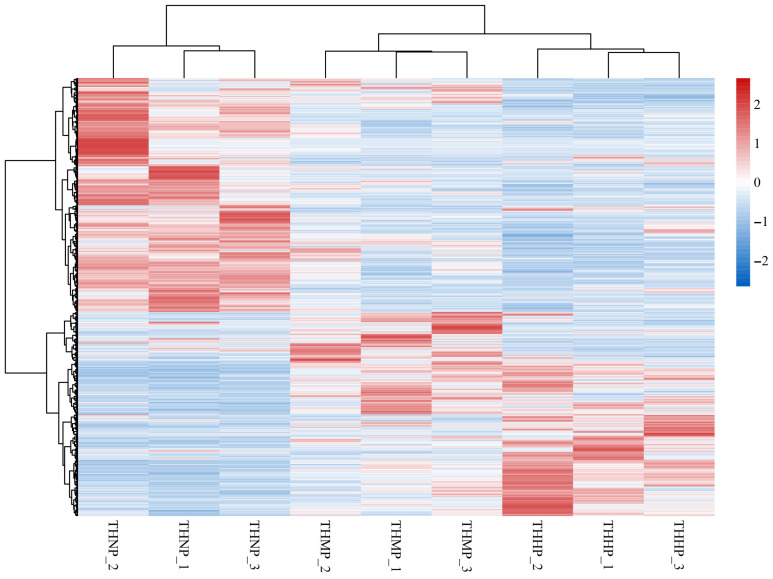
Cluster heatmap of DEG expression in oysters fed with organic selenium-supplemented feed. Horizontally, the heatmap represents different samples, while vertically, it represents various genes. The color transition from red to blue indicates gene expression from high to low.

Cluster heatmaps were utilized to demonstrate the correlation between samples and the expression of genes across different treatment groups (Figure 2). In this study, all the DEGs among the three comparison groups were used to create the differential gene cluster heatmap. The similar expression patterns of genes in the three samples of the same treatment method indicate a high correlation between the samples. The varied expression patterns of genes between different treatment groups highlight the significant impact of adding organic selenium to the feed on oysters.

**Figure 3:**
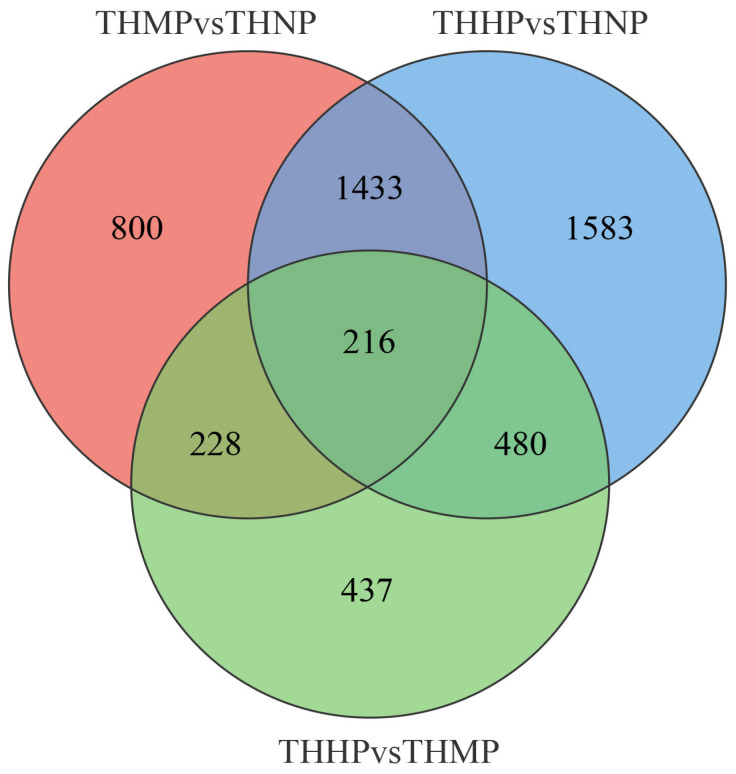
Venn diagram of DEG expression across different comparison groups after feeding with organic selenium. The intersecting areas represent the DEGs that exhibit significant differential expression across the various comparison groups.

Venn diagrams are used to show the specific number of DEGs among different groups (Figure 3). In this study, 216 DEGs exhibited significant differential expression across all the comparison groups. We hypothesize that these genes play a key role following the addition of organic selenium in oyster feed, and their specific functions warrant further investigation.

### 3.3. Functional Enrichment Analysis

Functional enrichment analysis of DEGs provides a clearer understanding of the specific biological functions that genes perform in oysters supplemented with organic selenium. In this study, we conducted Gene Ontology (GO) and KEGG functional enrichment analyses for DEGs exhibiting significant differences between various comparison groups.

**Figure 4:**
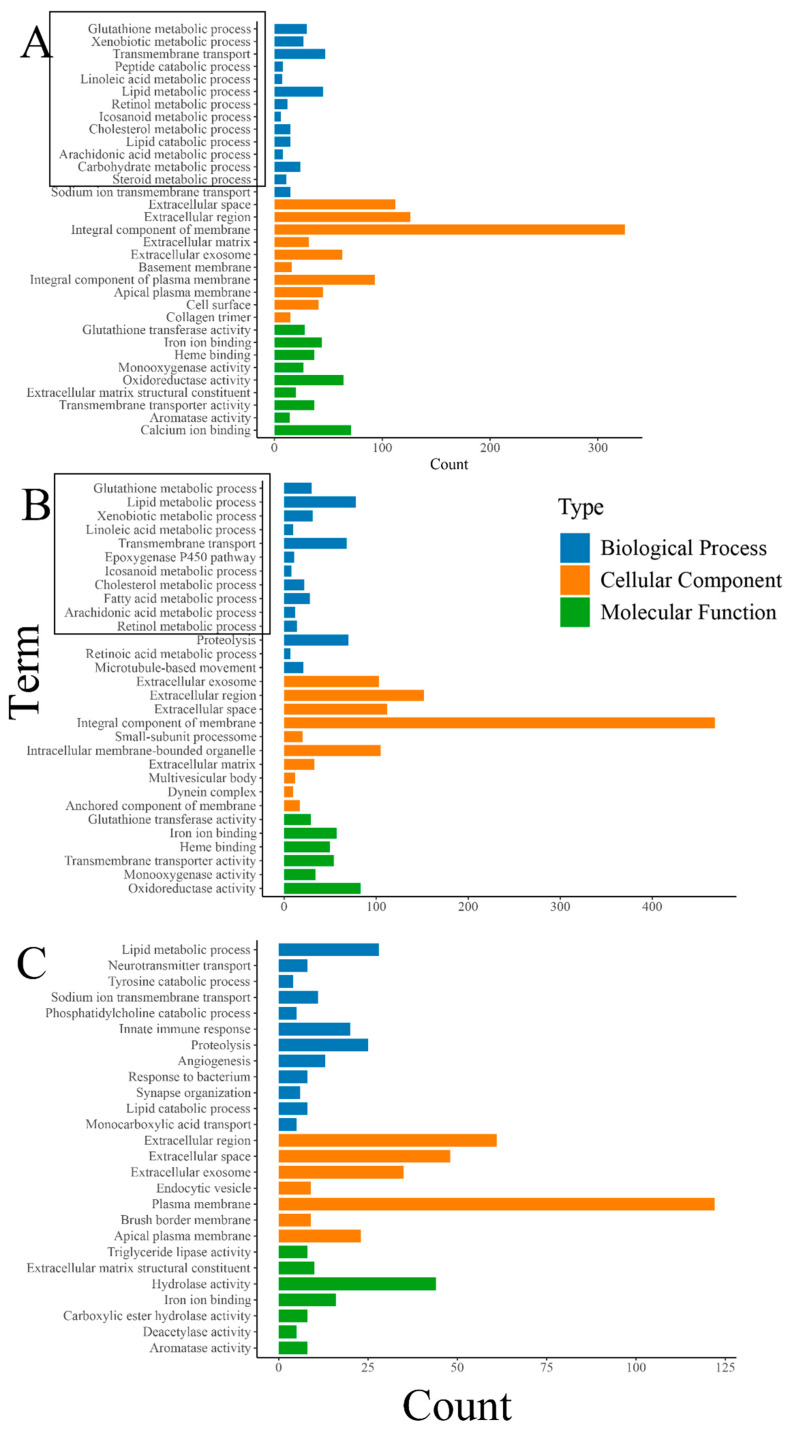
(**A**): GO enrichment analysis of DEGs between the treatment group with 2 ppm selenium (THMP) and the control group (THNP); (**B**): GO enrichment analysis of DEGs between the treatment group with 4 ppm selenium (THHP) and the control group (THNP); (**C**):GO enrichment analysis of DEGs between the treatment group with 2 ppm selenium (THMP) and the treatment group with 4 ppm selenium (THHP). The horizontal axis represents the number of genes, and the vertical axis represents different GO terms. Different colors denote different GO categories(with bold black indicating metabolism-related terms).

The results of the GO enrichment analysis revealed a significant enrichment of various metabolic-related biological processes, including “glutathione metabolic process”, “lipid metabolic process”, and “fatty acid metabolic process”. Also, biological processes closely related to the growth and development of organisms such as “transmembrane transport” were significantly enriched (Figure 4). The enrichment of numerous metabolic-related processes also indicates that feeding oysters with organic selenium significantly enhances their metabolic level, although this still requires specific validation through biochemical experiments. Differently, the enrichment analysis of DEGs between the treatment groups “THHP” and “THMP” showed significant enrichment of some immune and protein hydrolysis-related biological processes. This phenomenon might be due to the addition of organic selenium at a concentration of 4 ppm, possibly exceeding the optimal level for oysters, causing stress and triggering immune responses and other defensive processes.

**Figure 5:**
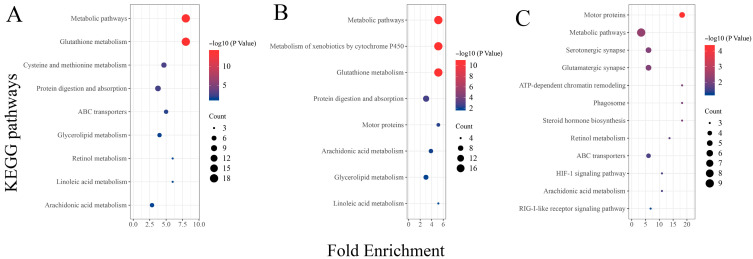
KEGG enrichment analysis of DEGs in different treatment groups of oysters fed with organic selenium. The horizontal axis represents the enrichment factor, and the vertical axis represents different KEGG signaling pathways. (**A**): KEGG enrichment analysis of DEGs between the treatment group with 2 ppm selenium (THMP) and the control group (THNP); (**B**): KEGG enrichment analysis of DEGs between the treatment group with 4 ppm selenium (THHP) and the control group (THNP); (**C**): KEGG enrichment analysis of DEGs between the treatment group with 2 ppm selenium (THMP) and the treatment group with 4 ppm selenium (THHP).

Analysis of the results from the KEGG functional enrichment analysis reveals, similarly to the GO enrichment results, a significant enrichment of metabolic-related signaling pathways between the two different concentration treatment groups (THMP and THHP) compared with the THNP group (Figure 5). These findings also confirm that feeding oysters with organic selenium significantly impacts the metabolic level of the organism. Additionally, DEGs between the different treatment groups (THHP and THMP) were primarily enriched in immune defense and other signaling pathways. This result suggests that a 4 ppm selenium concentration might not be optimal for oysters. However, the number of DEGs in this comparison group is relatively lower compared to others, indicating that this concentration may not be lethal but may activate certain immune defenses in oysters.

### 3.4. PPI Network Analysis

**Figure 6:**
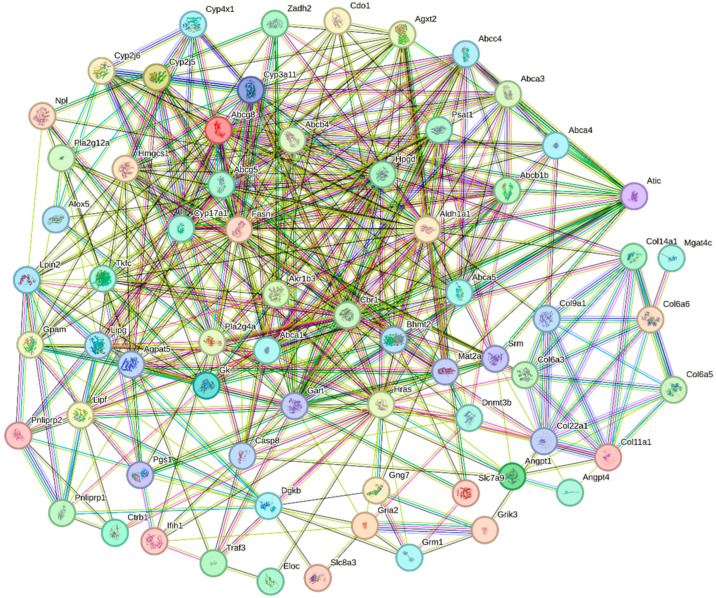
PPI analysis of DEGs involved in the KEGG signaling pathways in oysters after feeding with organic selenium. Each node represents a protein, and the lines between nodes represent interaction relationships.

In this study, we uploaded gene sequences of all the DEGs significantly enriched in the KEGG signaling pathways to the STRING website for homologous sequence alignment, using the sequences with the highest scores for the construction of a PPI network (Figure 6 and Table 3). Through a comprehensive analysis of the PPI and KEGG enrichment results, we identified twelve key DEGs that play a crucial role in oysters after being fed with organic selenium (Table 4). These DEGs are likely involved in promoting the growth and development of oysters following the addition of organic selenium to their feed, as suggested by their functional enrichment and expression patterns.

### 3.5. Quantitative Validation of DEGs

**Figure 7:**
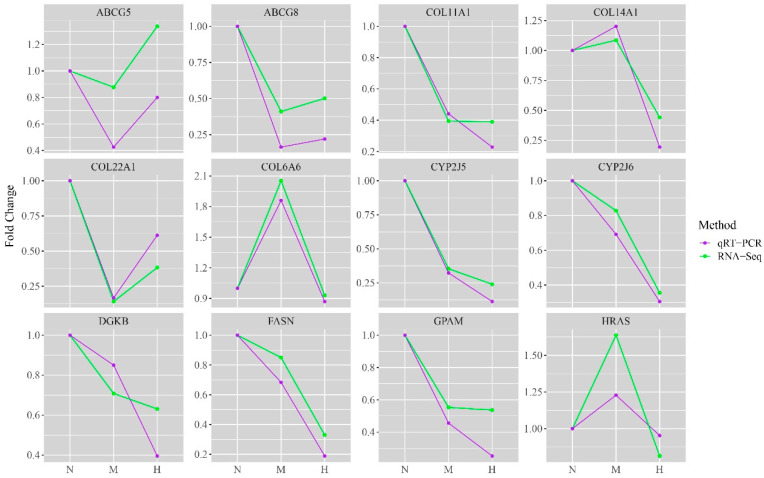
Quantitative validation of RNA-Seq. The expression trend of DEGs consistently proved the accuracy of the sequencing results. The vertical axis indicates the fold difference between treatment groups. The horizontal axis indicates the different groups: N—fold Change between THNP group and THNP group; M—fold Change between THMP group by THNP group; H—fold Change between THHP group and THNP group.

The fluorescence quantitative PCR was used to verify the expression levels of the key genes. The qRT-PCR results demonstrated that the expression trends of the key genes were consistent between different concentrations and that the sequencing results were reliable (Figure 7).

## 4. Discussion

### 4.1. Expression Analysis of DEGs

Selenium is an essential trace element required for the synthesis of various selenoproteins involved in numerous biological processes [30]. In this study, comparative transcriptomic analysis revealed notable differences in gene expression among the experimental groups with varying selenium concentrations. The results suggest that selenium at a concentration of 4 ppm has a greater impact on oysters compared to 2 ppm. The gene expression patterns observed in the volcano plots support this finding. Clustering analysis further showed consistent expression patterns within each treatment group and clear distinctions between groups, indicating good sample reproducibility and providing a solid foundation for subsequent bioinformatics analysis. 

### 4.2. Functional Enrichment Analysis of DEGs

Functional enrichment analysis helps clarify the biological processes in which DEGs are involved [31]. In this study, GO and KEGG enrichment analyses revealed that DEGs between the THNP and THMP groups were mainly associated with metabolic processes such as lipid and fatty acid metabolism, which are crucial for development and immunity [32]. The enrichment of these pathways suggests that selenium exposure affects oyster metabolism. Similar GO results were observed between THNP and THHP, but with more DEGs, indicating a stronger effect at 4 ppm. In contrast, DEGs between THMP and THHP were enriched in immune-related processes, implying an immune response under higher selenium levels. As the innate immune system plays a central role in maintaining internal homeostasis [33], the results suggest that a 4 ppm concentration of selenium may not be optimal for oyster growth and development, as it appears to induce immune-related responses. KEGG analysis supported these findings, with metabolism-related pathways enriched in both selenium-treated groups compared to THNP, and immune-related pathways significantly enriched between THMP and THHP. Notably, the RIG-I-like receptor signaling pathway, a key innate immune pathway, was significantly activated. As a critical member of the pattern recognition receptor (PRR) family, RIG-I-like receptors can initiate innate immune defenses through this pathway, thereby protecting the host organism [34]. These results suggest that appropriate selenium levels can enhance metabolism and promote growth, while higher concentrations (e.g., 4 ppm) may induce immune responses in oysters. 

### 4.3. Functional Analysis of Key DEGs

Proteins are the basis of life activities, and the growth and development of organisms cannot be separated from the support of proteins [35,36,37]. The PPI is able to analyze the interaction relationship between genes encoding proteins, which can help us to understand the molecular mechanism of DEGs encoding proteins more clearly [38]. In this study, we constructed a PPI network using DEGs significantly enriched in the KEGG signaling pathway. Based on the results of the PPI and KEGG enrichment analysis, the signaling pathways and DEGs that played a key role after selenium addition to oyster feed were identified.

#### 4.3.1. Glutathione Metabolism During Selenium Supplementation

Glutathione, an antioxidant synthesized by a two-enzyme reaction catalyzed by glutamate cysteine ligase and glutathione synthetase, plays a key role in protecting cells from oxidative damage and the toxicity of xenobiotic electrophilic reagents as well as maintaining redox homeostasis [39,40,41]. Usually, glutathione can be used directly as an antioxidant to protect cells from free radicals and pro-oxidants and as a cofactor for antioxidant and detoxification enzymes (glutathione peroxidase, glutathione S-transferase, and glyoxalase) [42,43,44]. A previous study has pointed out that tropical fish show a stress response specifically characterized by increased glutathione-S-transferase activity in the gills and increased concentration of reduced glutathione (GSH) in the liver after a high temperature stimulus [45]. This suggests that glutathione is effective in enhancing the antioxidant capacity of organisms and protects them from damage. Nguyen et al. conducted a metabolomic analysis of blood cells of Perna canaliculus after copper stimulation and found that the level of glutathione metabolism exhibits oxidative stress in organisms [46]. In the enrichment analysis results of this study, gene expression related to glutathione metabolism showed significant upregulation in the THMP and THHP groups compared to the THNP group. We found that the glutathione metabolism pathway was significantly upregulated, which may enhance the antioxidant capacity of oysters and thereby support their growth and fattening process, ultimately contributing to improved economic benefits in oyster culture.

#### 4.3.2. Collagen Family

Collagen is a structural protein containing three helical structural domains that play key roles in tissue renewal and repair [47,48,49]. It is primarily involved in forming fibrillar and microfibrillar networks in the extracellular matrix, basement membrane, and other extracellular structures [50,51,52]. In addition to maintaining tissue integrity, collagen participates in cell adhesion, wound healing, and inflammatory regulation [53,54]. In this study, several collagen family genes, including *COL11A1*, *COL14A1*, *COL22A1*, and *COL6A6*, were significantly downregulated in oysters fed selenium-enriched yeast. This downregulation may suggest potential effects of selenium supplementation on tissue remodeling or extracellular matrix dynamics. However, without direct phenotypic, histological, or health-related data, this remains a hypothesis, and further functional validation is required to confirm any biological impact. 

#### 4.3.3. Analysis of Hub Genes

Through the integrated analysis of PPI network and KEGG enrichment results, three key genes were identified: *FASN*, *HRAS*, and *ABCG5*. Fatty acids play essential roles in energy transport and storage, cell membrane structure, and hormone synthesis [55,56,57]. FASN, a key enzyme in lipid metabolism, is vital for growth and development [58,59]. In this study, FASN was significantly downregulated in the selenium-treated groups, possibly due to the inhibition of fatty acid metabolism by organic selenium. This metabolic suppression may contribute to oyster fattening. HRAS encodes H-Ras, a core component of the RAS/MAPK signaling pathway, which regulates cell proliferation, differentiation, survival, and apoptosis [60,61,62]. Here, HRAS expression showed an initial increase followed by a decrease with rising selenium concentration, suggesting that 2 ppm selenium may promote oyster cell proliferation, whereas higher concentrations may impair growth. Cholesterol is crucial for cell growth and function, and imbalances can lead to disease [63,64]. ABCG5 belongs to the ATP-binding cassette (ABC) transporter family. After heterodimerizing with its partner protein in the endoplasmic reticulum, it localizes to the apical membrane and participates in the transport of cholesterol and phytosterols, as well as bile secretion [65,66]. In this study, its expression decreased at 2 ppm selenium but increased again at 4 ppm, implying that moderate selenium enhances cholesterol levels to support growth, while excessive selenium may upregulate ABCG5 to reduce cholesterol, potentially affecting development. Notably, immune-related signaling pathways were enriched in the comparison between THMP and THHP, suggesting that 4 ppm selenium may be excessive for oysters and could trigger immune defense responses.

## 5. Conclusions

In this study, high-throughput transcriptome sequencing was employed to investigate the molecular mechanisms altered in oysters following dietary supplementation with different concentrations of selenium-enriched yeast. Through the quantitative analysis of DEGs, GO and KEGG functional enrichment analysis, and comprehensive analysis of protein–protein interaction networks, twelve key DEGs and metabolism-related signaling pathways were identified following selenium addition to oyster feed.

The downregulation of the glutathione metabolic pathway suggests that the addition of organic selenium can facilitate the fattening process of oysters to some extent. The downregulation of the collagen family also demonstrated that organic selenium had an effect on changes in tissue structure. The changes in the expression trends of the three pivotal genes also indicated that the addition of moderate amounts of organic selenium could significantly promote the fattening process of oysters, and thus improve the economic benefits of oyster farming. In summary, feeding appropriate concentrations of selenium-enriched yeast can help oysters accelerate the fattening process. The results of this study may provide a suitable reference for feed addition to oysters during aquaculture.

## Figures and Tables

**Table 1:** List of primers used for qRT-PCR validation.

Gene Name	Forward Primer (5′–3′)	TM (°C)	Reverse Primer (5′–3′)	TM (°C)	Amplicon Length (bp)
*ABCG5*	ACCGCTTCCTTCACAACCTGAC	60	CTGCCACATTCTTCAAACCCATCTC	58	141
*ABCG8*	CCCTACTTTACCTCCATTGGCTACC	59	TTTCGTCTCCCGCTCTGTTTCG	60	114
*COL11A1*	TTACACCGCTGCCTACCATAACTC	59	CCCTCCACCAACTGCCATTCC	62	94
*COL14A1*	CTTGAGCCCGACCAGACCATAAG	60	AACCCAGTGTCCCATCCCAATAAC	59	87
*COL22A1*	TGGACGGGACCTAATTTCACTGAC	59	TTCGGAGACCATTGTACGGCTTC	59	139
*COL6A6*	ACGGAGCTGTACGCCATTGC	61	ACCGATGCTTGGATCTAACTTGAGG	59	120
*CYP2J5*	GCGGCTCAGTTTACAAACAGACC	59	ACGGACCTTCTTCCAATGCTCTC	59	111
*CYP2J6*	GGAACAGAGACCACCGCAACC	61	ACTTCTCGCCCTAACCCAACAAC	59	119
*DGKB*	GATTGCTCGGGTCAGAGTTCATTTC	58	CATACACTTGGCGTGGATTCAGAAG	58	148
*FASN*	AGCCTTAGTGGACCTATTGAGAAGC	58	GTCAGGGAGCCATCAGCATACC	60	102
*GPAM*	AGAGACCAGCAGCGATACCAATG	59	CCACCTGACTGACGGGCATAC	61	147
*HRAS*	CTATGCGAGACCAGTACATGAGGAC	59	ACCCACCAACACCATAGGAACTTC	59	149

**Table 2:** RNA-Seq results.

Sample	Total Reads	Total Map	Raw Reads	Clean Reads	Error Rate	Q20	Q30
THNP_1	43,508,618	29,437,606 (67.66%)	46,061,122	43,508,618	0.02	98.24	94.59
THNP_2	39,963,598	29,136,484 (72.91%)	42,560,702	39,963,598	0.03	97.88	94.04
THNP_3	40,757,216	28,651,116 (70.30%)	42,621,702	40,757,216	0.02	97.97	94.21
THMP_1	45,389,286	31,569,508 (69.55%)	47,526,686	45,389,286	0.03	97.28	92.24
THMP_2	44,102,584	29,583,679 (67.08%)	46,221,506	44,102,584	0.03	95.91	89.45
THMP_3	44,125,492	31,220,930 (70.75%)	46,475,360	44,125,492	0.03	97.37	92.46
THHP_1	45,734,110	31,778,440 (69.49%)	48,218,282	45,734,110	0.03	97.42	92.57
THHP_2	43,037,580	32,118,082 (74.63%)	44,728,006	43,037,580	0.02	97.98	94.24
THHP_3	42,060,542	30,535,558 (72.60%)	43,134,446	42,060,542	0.03	97.83	93.85

**Table 3:** Protein interaction network related parameters.

Network Stats	
Number of nodes	67
Number of edges	439
Average node degree	13.1
Clustering coefficient	0.531
Expected number of edges	199
PPI enrichment *p*-value	1 × 10^−16^

**Table 4:** Number of PPI and KEGG of twelve key DEGs.

Gene ID	Gene Name	Node Degrees of PPI	Number of KEGG
*ABCG5*	ATP binding cassette subfamily G member 5	25	1
*ABCG8*	ATP binding cassette subfamily G member 8	23	1
*COL11A1*	collagen type XI alpha 1 chain	8	2
*COL14A1*	collagen type XIV alpha 1 chain	9	1
*COL22A1*	collagen type XXII alpha 1 chain	10	2
*COL6A6*	collagen type VI alpha 6 chain	8	2
*CYP2J5*	cytochrome P450, family 2, subfamily j, polypeptide 5	15	2
*CYP2J6*	cytochrome P450, family 2, subfamily j, polypeptide 6	12	3
*DGKB*	diacylglycerol kinase beta	12	1
*FASN*	fatty acid synthase	39	1
*GPAM*	glycerol-3-phosphate acyltransferase, mitochondrial	13	1
*HRAS*	HRas proto-oncogene, GTPase	30	1

## Data Availability

Data will be made available upon request.

## Supplementary Materials

The following supporting information can be downloaded at: https://www.mdpi.com/article/10.3390/biology14070898/s1, Table S1: Experimental oyster phenotypic data in 2023.04.

## Abbreviations

The following abbreviations are used in this manuscript:

*C. gigas*	*Crassostrea gigas*
GO	Gene Ontology
KEGG	Kyoto Encyclopedia of Genes and Genomes
DEGs	Differentially expressed genes
qRT-PCR	quantitative Reverse Transcription Polymerase Chain Reaction
PPI	Protein–Protein Interaction
