# Supplementary material for: Metabolic Alterations in Crassostrea Gigas After Feeding Selenium-Enriched Yeast Based on Transcriptomic Analysis
Source: Biology (Basel). 2025 Jul 21;14(7):898. doi: 10.3390/biology14070898 (PMC12292104; doi:10.3390/biology14070898)
Supplement: Supplementary file 1 [file biology-14-00898-s001.zip › biology-3748924-supplementary.pdf]

Table S1. Experimental oyster phenotypic data in 2023.04

clusters	serial number	High(mm)	Long(mm)	Width(mm)
THNP	1	72.43	48.36	28.80
	2	70.45	52.66	27.31
	3	67.61	49.47	29.62
	4	64.27	56.77	28.20
	5	63.87	50.59	26.79
	6	81.69	57.67	25.61
	7	74.34	50.12	25.33
	8	66.58	46.68	27.73
	9	77.38	49.95	28.98
THMP	1	76.63	51.71	33.56
	2	69.98	52.28	25.33
	3	65.14	37.18	31.93
	4	65.63	51.56	30.98
	5	81.15	48.66	24.24
	6	65.73	47.07	27.67
	7	87.13	52.16	30.27
	8	74.22	45.90	25.49
	9	74.28	52.40	21.37
THHP	1	71.09	50.09	23.40
	2	76.16	48.28	29.70
	3	74.66	57.84	33.08
	4	66.86	45.33	23.30
	5	74.03	46.68	26.42
	6	62.34	47.57	21.54
	7	69.33	48.91	36.17
	8	81.12	55.21	25.26
	9	71.46	50.06	24.31

Experimental oyster phenotypic data in 2023.05

clusters	serial number	High(mm)	Long(mm)	Width(mm)
THNP	1	78.26	48.31	30.54
	2	66.69	53.01	29.95
	3	68.30	57.91	31.73
	4	77.37	48.24	20.44
	5	72.32	54.55	28.19
	6	67.15	44.97	24.09
	7	68.29	50.12	26.03
	8	65.76	50.48	22.33
	9	74.47	55.19	24.45
THMP	1	89.46	49.65	33.13
	2	65.70	50.73	19.85

	3	79.70	59.63	34.05
	4	77.04	51.41	34.08
	5	72.04	53.38	27.39
	6	70.28	51.25	25.47
	7	74.49	52.84	27.58
	8	62.90	40.95	23.85
	9	77.22	46.37	26.30
THHP	1	67.60	59.09	26.59
	2	70.37	49.66	35.35
	3	66.30	47.44	27.08
	4	68.92	48.48	34.99
	5	73.64	50.75	20.92
	6	81.28	56.44	32.75
	7	67.64	51.64	27.92
	8	76.54	51.31	25.80
	9	71.68	50.23	22.34

Experimental oyster phenotypic data in 2023.06

clusters	serial number	High(mm)	Long(mm)	Width(mm)
THNP	1	75.88	53.23	29.39
	2	67.39	48.29	25.09
	3	77.21	48.40	23.00
	4	67.32	61.36	30.83
	5	74.40	56.52	25.94
	6	75.64	46.31	25.21
	7	67.01	52.10	25.77
	8	70.72	52.24	27.53
	9	62.88	52.12	22.24
THMP	1	62.21	47.97	24.36
	2	66.05	47.25	26.50
	3	72.68	49.89	25.86
	4	82.38	44.38	28.83
	5	67.17	51.62	27.63
	6	86.89	49.00	23.40
	7	68.95	53.91	25.87
	8	78.64	63.16	35.16
	9	61.69	52.70	22.59
THHP	1	72.48	60.85	25.94
	2	73.95	46.27	27.71
	3	59.96	46.49	26.41
	4	63.13	50.95	22.96
	5	71.51	49.36	27.65
	6	79.09	43.20	26.71
	7	63.32	48.39	23.25

	8	78.29	51.65	25.33
	9	75.96	46.29	28.95
